# Trophic downgrading reduces spatial variability on rocky reefs

**DOI:** 10.1038/s41598-020-75117-2

**Published:** 2020-10-22

**Authors:** Matthew S. Edwards, Brenda Konar

**Affiliations:** 1 Department of Biology, San Diego State University, 5500 Campanile Dr., San Diego, CA 92182 USA; 2 Institute of Marine Science, University of Alaska Fairbanks, Fairbanks, USA

**Keywords:** Biogeography, Ecosystem ecology

## Abstract

Trophic downgrading in coastal waters has occurred globally during recent decades. On temperate rocky reefs, this has resulted in widespread kelp deforestation and the formation of sea urchin barrens. We hypothesize that the intact kelp forest communities are more spatially variable than the downgraded urchin barren communities, and that these differences are greatest at small spatial scales where the influence of competitive and trophic interactions is strongest. To address this, benthic community surveys were done in kelp forests and urchin barrens at nine islands spanning 1230 km of the Aleutian Archipelago where the loss of predatory sea otters has resulted in the trophic downgrading of the region’s kelp forests. We found more species and greater total spatial variation in community composition within the kelp forests than in the urchin barrens. Further, the kelp forest communities were most variable at small spatial scales (within each forest) and least variable at large spatial scales (among forests on different islands), while the urchin barren communities followed the opposite pattern. This trend was consistent for different trophic guilds (primary producers, grazers, filter feeders, predators). Together, this suggests that Aleutian kelp forests create variable habitats within their boundaries, but that the communities within these forests are generally similar across the archipelago. In contrast, urchin barrens exhibit relatively low variability within their boundaries, but these communities vary substantially among different barrens across the archipelago. We propose this represents a shift from small-scale biological control to large-scale oceanographic control of these communities.

## Introduction

Trophic downgrading occurs when apex predators have been extirpated over large geographic regions, which can lead to important consequences for ecosystem functioning due to both direct and indirect cascading effects^1^. This has been observed globally across a variety of terrestrial and marine ecosystems^2–7^. Often, trophic downgrading triggers increases in herbivore populations, thereby changing overall community structure^6,8,9^ and altering patterns of ecosystem productivity^10–12^. Trophic downgrading can be especially important if it ultimately affects ecosystem engineers that provide habitat, which modifies the physical environment, regulates primary production and energy flow, and generally supports high biodiversity. For example, the extirpation of gray wolves from Yellowstone National Park, USA in the early 1900s resulted in reduced predation on elk and increased herbivory on forest-forming trees^13^. This ultimately led to changes in the morphology and hydrology of the region’s river systems and its riparian plant communities^14,15^. Similarly, the loss of sea otters from the nearshore habitats of the Aleutian Archipelago during the 1980s and 1990s resulted in reduced predation on herbivorous sea urchins and a subsequent overgrazing of the regions kelp forests^2^. This led to reduced biodiversity^16^, altered food web dynamics^17^, and reduced benthic ecosystem productivity^12^ in the coastal environment. Further, when a community has been downgraded, its resilience (i.e., recovery and stability after a disturbance) can decrease in comparison to intact communities^3^. This has been an important consideration in the design of marine protected areas (MPAs) and terrestrial parks and reserves, which typically protect apex predators to maintain diversity and normal ecosystem functioning^7,18–20^.

Spatial and temporal variability in community structure are important components of ecological systems, and understanding how variability changes in space and time can infer a wide range of ecological processes^21–27^. For example, resistance to biological invasions is strongly correlated with variability in environmental conditions^28^ and community structure^29^, with less variable communities being more resistant to invasion than highly variable communities. However, the loss of foundation species can increase susceptibility to biological invasions^30,31^. Moreover, patterns of variability can themselves change at different temporal and spatial scales coincident with environmental and demographic forcing factors^21,25,26,32,33^. Indeed, Levin^21^ noted that the problem of pattern and scale is the central problem in ecology and that it is important to find ways to quantify patterns of variability in space and time, understand how patterns change with scale, and understand the causes and consequences of pattern. This can be especially important in ecosystems where different forcing factors affect communities across a range of spatial scales^33–35^.

Kelp forests are benthic, biogenic habitats that include highly productive primary producers rivaling those of cultivated agricultural fields and tropical rainforests in productivity^36–39^. This productivity and the associated formation of complex, three-dimensional biogenic habitat enhances local biodiversity and secondary productivity^16,40,41^. However, kelp forests in many areas of the world have been trophically downgraded as their apex predators have been removed^42,43^. For example, in Tasmania, the loss of predatory lobsters has led to increases in sea urchin abundance and an increased risk of catastrophic shifts to widespread sea urchin barrens^44^. Such deforestation of kelp forests due to sea urchin grazing is becoming more common in mid-latitudes^45–47^ (see also citations in Steneck et al.^9^). This generally results in a loss of biodiversity and associated ecosystem services, as kelp forests generally support more species^16,48^ (this study) and exhibit greater primary productivity and habitat complexity^12,49,50^ than sea urchin barrens. Consequently, we expected there would be more combinations of species that could spatially differentiate the kelp forest communities than the sea urchin barren communities. This would be especially important at small spatial scales where the influence of biological interactions (e.g., competition, grazing, and predation) in spatial structuring of communities can be strongest and potentially obscure large-scale variability due to climate or oceanographic variability^51–55^.

**Figure 1:**
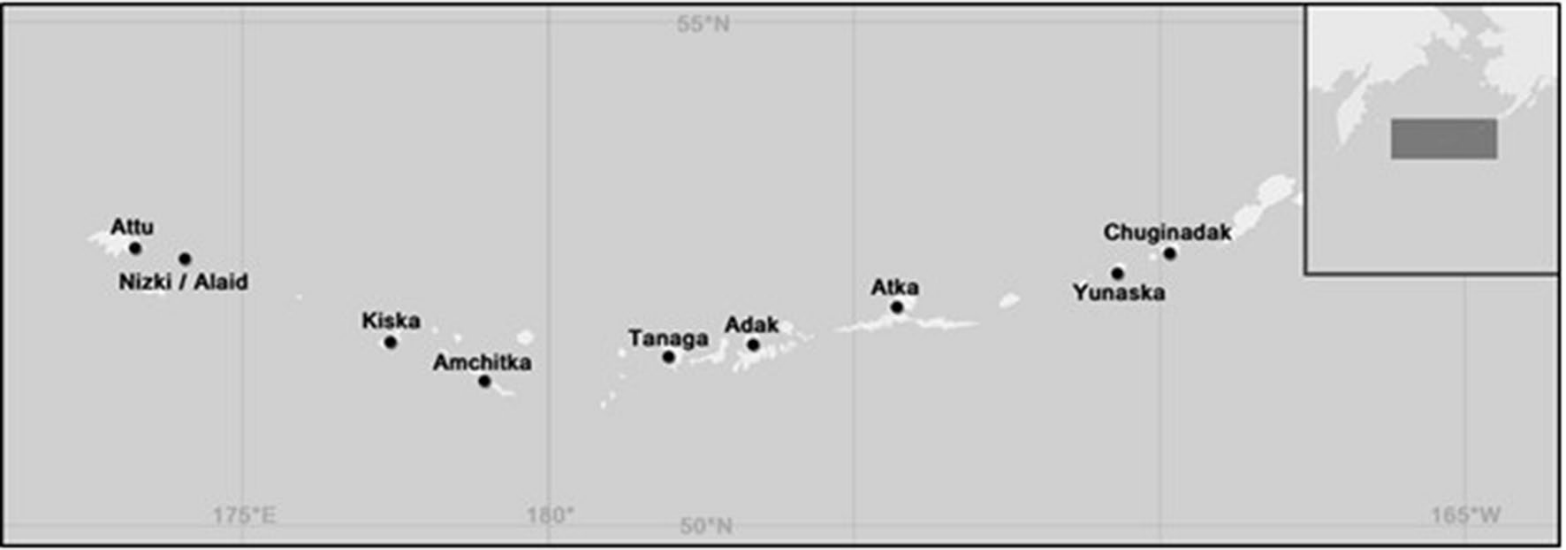
Map of study area showing the nine islands sampled across the Aleutian Archipelago (inset shows portion of archipelago where islands are located). Coordinates in decimal degrees for approximate sampling locations are: Attu: 52.92°, 173.20°; Nizki/Alaid: 52.74°, 174.00°; Kiska: 51.97°, 177.58°; Amchitka: 51.41°, 179.28°; Tanaga: 51.81°, − 177.94°; Adak: 51.87°, − 176.66°; Atka: 52.10°, − 174.69°; Yunaska: 52.66°, − 170.74°; and Chuginadak: 52.84°, − 169.75°. Image from Metzger et al. 2019.

The shallow subtidal regions of the Aleutian Archipelago (Fig. 1) are a well-studied simple system under top-down control^56^. Here, sea otters that were previously hunted to near extinction by the fur trade began to recover in the early 1900s^57^. This recovery continued through the 1980s when the removal of sea otters by killer whales became apparent by the 1990s^56,57^. Following the removal of otters, the abundance and biomass of their primary prey, herbivorous sea urchins, dramatically increased^2^. These hyper-abundant sea urchins quickly overgrazed the macroalgal communities, causing widespread deforestation of the region’s kelp forests, and increases in the prevalence and extent of urchin barrens. These urchin barrens are largely devoid of all fleshy macroalgae but instead are dominated by sea urchins and coralline red algae^12,16^. They can be of varying ages depending on the timing of sea otter recovery and subsequent sea otter removal, but once formed they are stable over periods of at least several years^58^. Here, we use this trophically-mediated deforestation to examine spatial patterns of community variability in kelp forests and urchin barrens throughout the Aleutian Archipelago. We ask if non-downgraded communities with more species (i.e., intact kelp forests) exhibit greater spatial variability compared to downgraded communities with fewer species (i.e., urchin barrens)^16^. We then compare how these patterns of variability are distributed across different spatial scales, from meters to hundreds of kilometers. We do this for the whole communities and for different trophic guilds (i.e., primary producers, grazers, filter feeders, and predators). Based on previous observations we have made during numerous visits to the archipelago, we hypothesized that (1) the community state with higher biodiversity (intact kelp forests) will have greater overall spatial variability in community structure than the community state with lower biodiversity (downgraded urchin barrens), (2) patterns of variability will be greatest at small spatial scales for the kelp forests but greatest at large spatial scales for the urchin barrens, and (3) these patterns will be consistent among different trophic guilds.

## Results

**Figure 2:**
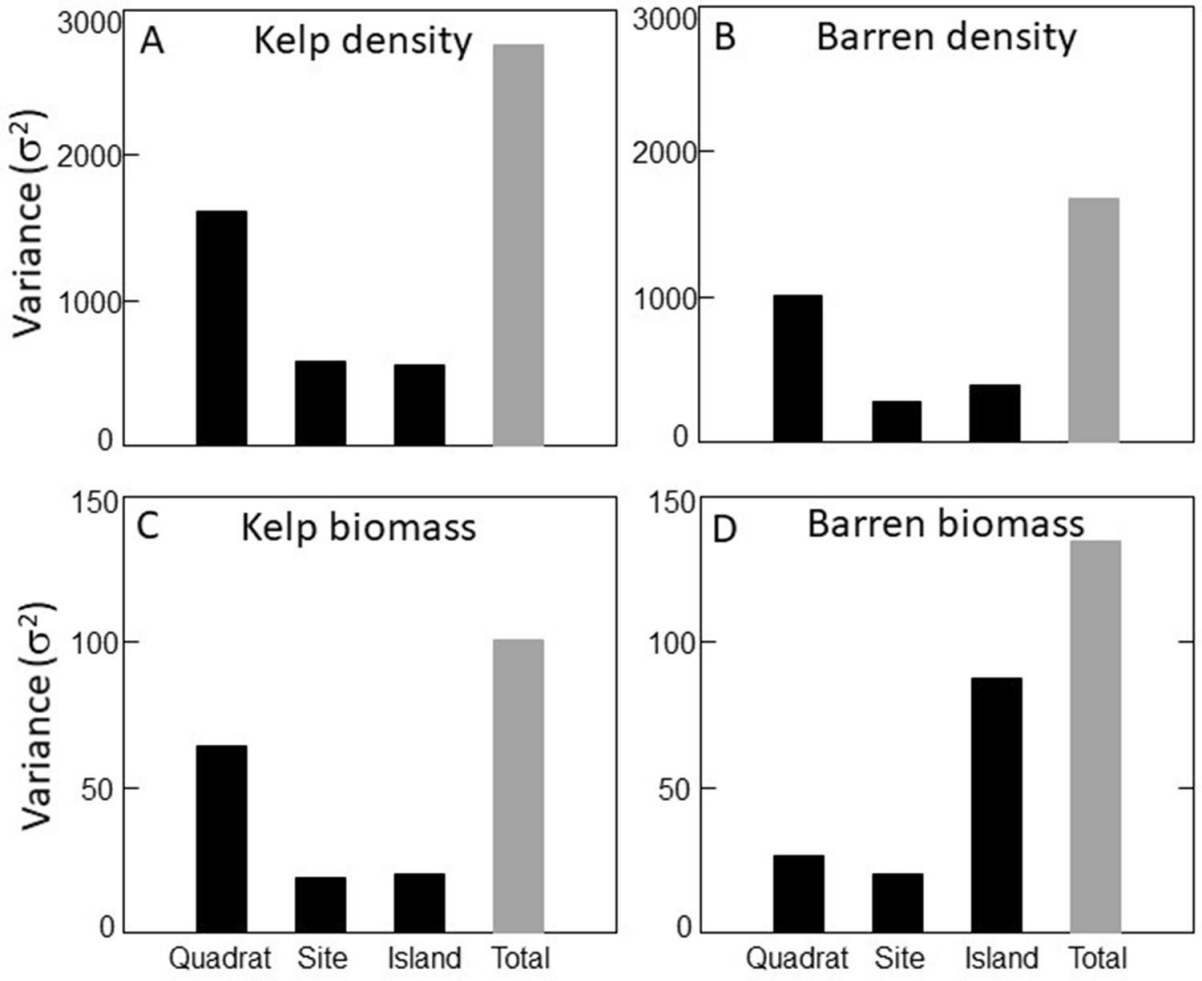
Bar graphs showing variance components (σ^2^) associated with each spatial scale in the PERMANOVA models for both density-based and biomass-based data. Gray bars on the right show total amount of variation in each model.

**Figure 3:**
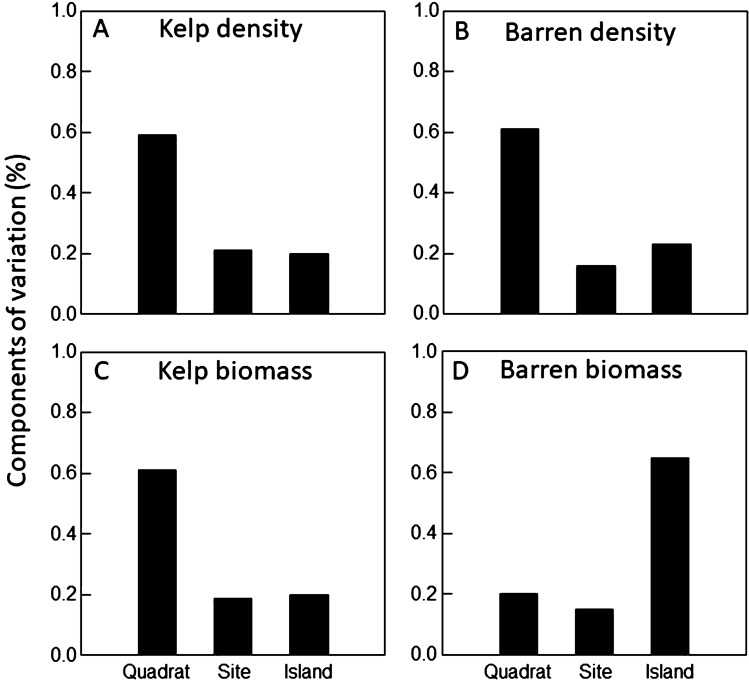
Bar graphs showing magnitude of effects (% of total variation) that is associated with each spatial scale in each PERMAOVA model for both density-based and biomass-based data.

**Figure 4:**
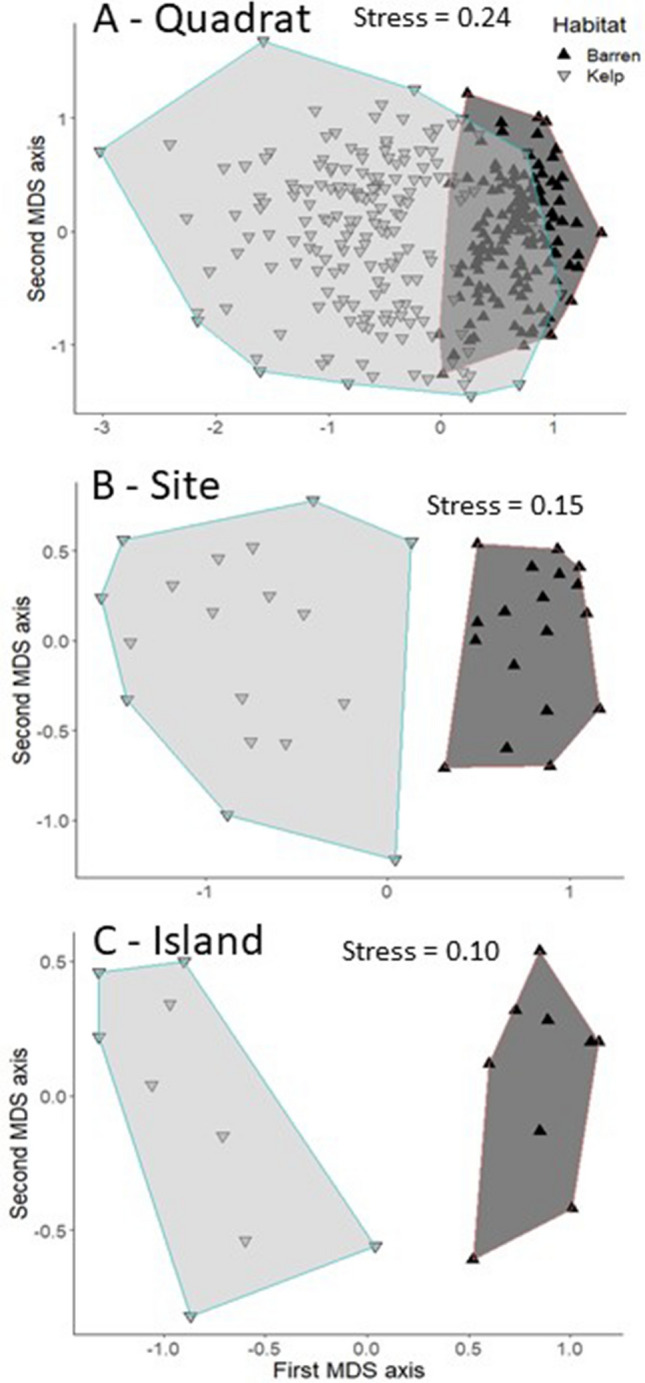
Density data-based nMDS plots comparing relative similarities in barren and kelp forests communities at different spatial scales; (**A**) among quadrats within each site, (**B**) among sites within each island, and (**C**) among islands. Data were square root transformed prior to analyses, and each resemblance matrix was based on zero inflated Bray–Curtis similarities. Shaded areas represent two-dimensional convex hulls representing areas connecting exterior data points.

**Figure 5:**
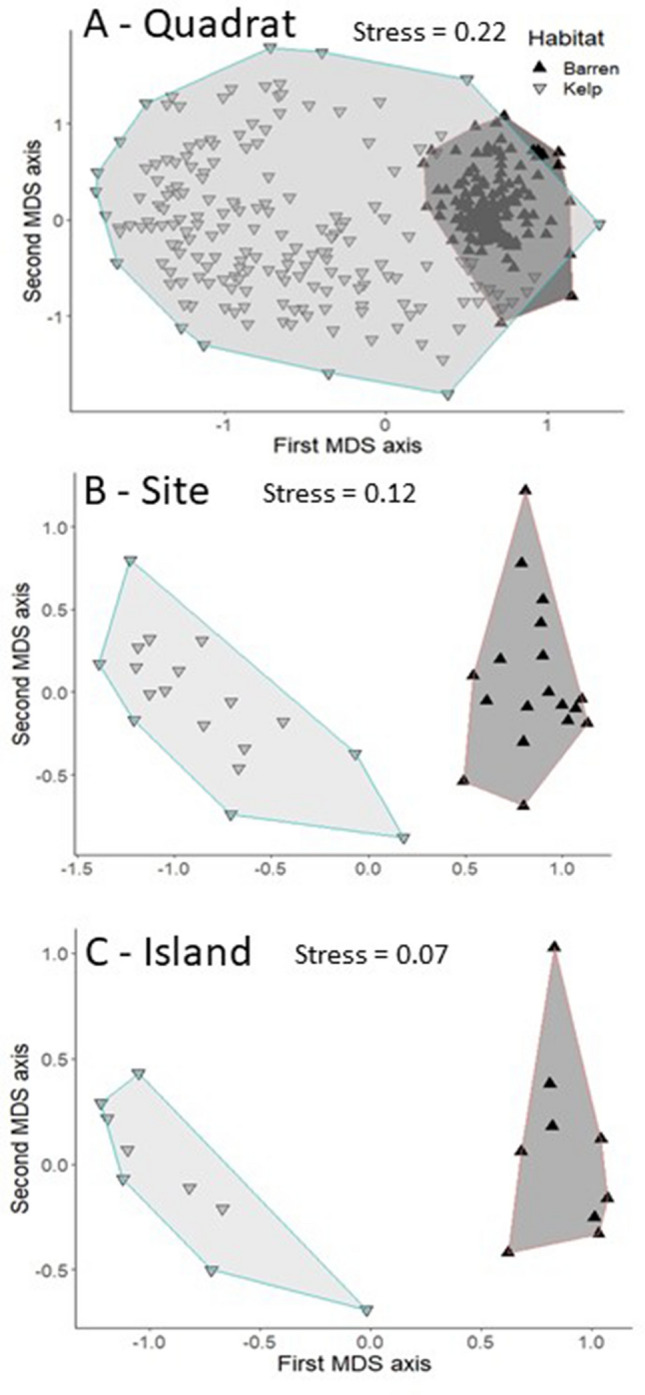
Biomass data-based nMDS plots comparing relative similarities in barren and kelp forests communities at different spatial scales; (**A**) among quadrats within each site, (**B**) among sites within each island, and (**C**) among islands. Data were square root transformed prior to analyses, and each resemblance matrix was based on zero inflated Bray–Curtis similarities. Shaded areas represent two-dimensional convex hulls representing areas connecting exterior data points.

We identified a total of 217 species in the kelp forests and 153 species in the urchin barrens using both density and biomass based data. Overall, both data types indicated that benthic community composition varied significantly among the nine islands, and between the two sites within each island in both the kelp forests and urchin barrens (Supplementary Table S1). Further, we found 1.6 times more total spatial variation in community structure in the kelp forests than the urchin barrens when using density data, but 1.3 times more total spatial variation in the urchin barrens when using biomass data (Fig. 2). This variation generally followed a scaling pattern within the kelp forests, with the largest amount of variation (59–61%) in the PERMANOVA models observed at the smallest spatial scale (i.e., among quadrats within each site), while much less of the variation (19–20%) was observed at the largest scale (i.e., among islands) (Fig. 3). Variation at the intermediate scale (i.e., among sites within each island) was similar to that of the largest scale, accounting for approximately 19–21% of the variation in the models. In contrast, when these patterns were examined within the urchin barrens, the amount of variation associated with each spatial scale in our PERMANOVA models depended on the type of data used. Here, the patterns were similar to those in the kelp forests when density data were used, with most of the variation in the model (61%) observed at the smallest spatial scale, and much less of the variation observed at the intermediate (16%) and largest (23%) scales (Fig. 3). In contrast, these patterns followed the opposite scaling pattern in the urchin barrens when biomass data were used. Specifically, the largest amount of variation (65%) in the PERMANOVA model was observed at the largest scale, while much less of the variation was observed at the intermediate (15%) and smallest (20%) scales (Fig. 3). Consequently, the kelp forest communities were 1.6 times more variable than the urchin barren communities at the smallest scale (among quadrats within each site), 2.1 times more variable at the intermediate scale (among sites within each island), and 1.4 times more variable at the largest scale (among islands) when based on density data (Figs. 2, 4). However, when based on biomass data, the kelp forest communities were 2.3 times more variable than the urchin barren communities at the smallest scale, but they were approximately the same at the intermediate scale, and the urchin barren communities were 1.3 times more variable than the kelp forest communities at the largest scale (Figs. 2, 5). It is important to note, however, that these components of variation are not independent of each other, as they are conditional on both the underlying amount of total variation in each PERMANOVA model and by each factor’s degrees of freedom within the model^59^, and care should therefore be taken when comparing them among PERMANOVA models.

Independent estimates of variability in community structure at each spatial scale were determined for each data type based on analyses of multivariate dispersions^60^. Doing this revealed that the kelp forest communities were more variable than the urchin barrens, and variation in kelp forest community structure followed the same scaling pattern as observed in the PERMANOVA models. In particular, for both density and biomass-based data, the greatest amount of variability in kelp forest community composition was observed at the smallest scale (among quadrats within each site) (MvDisp = 1.281 and 1.403, respectively), followed by the intermediate scale (among sites within each island) (MvDisp = 1.233 and 1.053, respectively), and then by the largest scale (among islands) (MvDisp = 1.205 and 1.024, respectively) (Table 1). In contrast, variation in community composition within the urchin barrens followed the opposite scaling pattern, with the greatest amount of variation observed at the largest scale for both density and biomass data (MvDisp = 0.795 and 0.976, respectively), followed by the intermediate scale (MvDisp = 0.767 and 0.947, respectively), and then by the smallest scale (MvDisp = 0.728 and 0.606, respectively) (Table 1). This resulted in the communities within the kelp forests being about 1.8 and 2.3 times more variable than those in the urchin barrens when examined at the smallest scale using density and biomass data, respectively. When examined at the intermediate scale, the communities in the kelp forests were 1.6 and 1.1 times more variable than those in the urchin barrens, respectively. At the largest scale, they were 1.5 and 1.1 times more variable than in the urchin barrens, respectively (Table 1).

**Table 1:** Multivariate dispersions (variances) in the composition of whole communities, and primary producer, grazer, filter feeder and predator assemblages observed in each habitat type (kelp forests and urchin barrens) at each spatial scale (quadrats, sites, and islands) for both (A) density-based and (B) biomass-based data. Data were square root transformed prior to analysis, and each resemblance matrix was based on zero inflated Bray–Curtis similarities.

Habitat	Scale	Communities	Primary producers	Grazers	Filter feeders	Predators
**(A) Density data**						
Kelp	Quadrat	1.281	1.499	1.176	1.289	1.200
	Site	1.233	1.466	1.151	1.377	1.125
	Island	1.205	1.467	1.072	1.457	1.068
Barren	Quadrat	0.728	0.512	0.828	0.717	0.805
	Site	0.767	0.534	0.849	0.623	0.875
	Island	0.795	0.533	0.928	0.543	0.932
**(B) Biomass data**						
Kelp	Quadrat	1.403	1.440	1.115	1.391	1.291
	Site	1.053	0.660	1.070	0.768	0.745
	Island	1.024	0.618	1.076	0.715	0.713
Barren	Quadrat	0.606	0.570	0.888	0.618	0.716
	Site	0.947	1.340	0.930	1.232	1.255
	Island	0.976	1.382	0.924	1.285	1.287

Multivariate dispersions were estimated using the MvDisp routine in Primer-E.

When patterns of variability were examined for the different trophic guilds (primary producers, grazers, filter feeders, and predators), similar patterns to those of the whole-communities were identified. First, we observed more species of primary producers (11 vs. 6), grazers (15 vs. 13), filter feeders (30 vs. 26), and predators (52 vs. 40) in the kelp forests than in the urchin barrens respectively, when using density data, though the differences in overall species numbers were only marginally significant (paired t test: t = 2.644, df = 3, P = 0.077). Likewise, we observed more species of primary producers (66 vs. 36), herbivores (18 vs. 12), filter feeders (71 vs. 54), and predators (54 vs. 45) in the kelp forests than in the urchin barrens, respectively, when using biomass data, but the differences in overall species numbers were again only marginally significant (paired t test: t = 2.89, df = 3, p = 0.063). Second, when density data were used, the assemblages making up the different trophic guilds were each more variable within the kelp forest than the urchin barrens at all spatial scales examined (Table 1). Third, primary producer, grazer, and predator densities within the kelp forests were each most variable at the smallest spatial scale and this variability decreased at the larger scales (Table 1). In contrast, primary producer, grazer, and predator densities within the urchin barrens followed the opposite pattern in that they were all least variable at the smallest scale and this variability increased at the larger spatial scales. The filter feeders, however, were an exception. Within the kelp forests, filter feeder densities were most variable at the largest scale and least variable at the smallest scale, but these followed the opposite pattern within the urchin barrens (Table 1). When biomass data were used, primary producer, grazer, filter feeder, and predator assemblages within the kelp forests were all most variable at the smallest scale (among quadrats within each site) and this variability decreased at the larger scales, while the opposite pattern was again observed within the urchin barrens (Table 1). Further, biomass of the assemblages belonging to the different trophic guilds were all more variable within the kelp forests than within the urchin barrens at the smallest scale, but they were generally more variable within the urchin barrens at the larger scales (Table 1). The exception to this were the grazers, which were more variable within the kelp forests than the urchin barrens at all spatial scales.

## Discussion

Trophically downgraded ecosystems are becoming more common worldwide, which is leading to conservation concerns due to the associated losses of biodiversity, productivity, and community resilience^1,9^. Downgrading often results in the formation of alternate stable states^4,6,8^ in which herbivores become hyper-abundant and autotrophs become rare. When this affects the distribution and abundance of ecosystem engineers, such as forest-forming trees and kelps that create habitat and supply energy for their communities, ecosystem function can be altered^12^ and biodiversity reduced^16^. Such ecosystem changes, however, can vary among different spatial scales due to a variety of forcing factors^21,24,32,33^. Here, we demonstrate that the intact kelp forests in the Aleutian Archipelago have more species than the trophically downgraded urchin barrens, and that this is consistent using both biomass and density data. Further, the intact kelp forest communities were most variable at the smallest spatial scale examined (among quadrats within each forest) and least variable at the largest spatial scale (among islands). This suggests that kelp forests create a variable habitat within their boundaries with high potential for species interactions (e.g., competition, grazing, predation), which are important drivers of small-scale variability^54,55^ and which can mask the influence of large-scale climate or oceanographic factors^51–53^. These habitat characteristics are then repeated over larger spatial scales (i.e., different kelp forests typically contain similar species assemblages and biological interactions), which reduces larger-scale variability.

Following trophic downgrading that resulted in reduced biodiversity as the kelp forests transitioned to urchin barrens^16^, the number of potential species interactions also declined. Although the timing of these changes varied among islands, the actual changes were consistent across the archipelago, which led to a transformation of the landscape^61^. This also led to changes in spatial variability in community structure and how it was distributed among the different spatial scales, but this depended on the type of data used. Specifically, when species abundances were estimated based on their densities, there was an overall reduction in total spatial variability in community structure at each spatial scale, and the barrens remained most variable at the smallest spatial scale and least variable at the larger spatial scales. In contrast, when species abundances were estimated based on their biomasses, variability in community structure again decreased at the smallest spatial scale, but it increased substantially at the larger spatial scales. This resulted in the urchin barren communities being least variable at the smallest spatial scale and most variable at the largest spatial scale. It also resulted in the urchin barrens exhibiting more total spatial variability in community structure than the kelp forests. These differences between the two data types appeared largely due to how each estimated the abundance of filter feeders and grazers (discussed below). Regardless, we believe this increase in large-scale variability reflected variation in oceanographic, topographic and hydrodynamic conditions, which can mask the importance of small-scale biological influences^33,62–64^.

Environmental conditions can vary among the islands that make up the Aleutian Archipelago. For example, island size and coastline morphology, differences in the dimensions and depths of the many oceanic passes that separate them, their associated shelf bathymetries, and their water mass properties can also influence the environment^65^. Currents influencing water masses in this region are complex. In the central and western Aleutians, the Alaska Stream flows westward along the Aleutian shelf break, providing Bering Sea in flow from Samalga Pass to Near Strait^66,67^. These waters are relatively cool, saline, and nutrient rich^66^. In contrast, the Aleutian North Slope Current flows along the Bering side of the islands from Amchitka Pass eastward^68,69^. These waters become warmer, fresher, and more nutrient poor to the east due to the influence of the Alaska Coastal Current^67^. An example of the complexities of the Aleutian environment can be seen at Samalga Pass, a well-described biogeographic break for some cold-water corals, zooplankton, demersal fishes, seabirds, and deep marine faunal communities^65^, especially for benthic invertebrate and macroalgal assemblages on coastal rocky reefs^70^. We propose that these large-scale oceanographic influences play a larger role in structuring the trophically downgraded urchin barrens, while prior to downgrading, the Aleutian kelp forest were presumably locally controlled by biological interactions.

When the different taxa within the two habitats were organized into their respective trophic guilds, we found similar results to those observed for the whole communities, with the exception of filter feeders. In particular, when abundances were estimated using density data, each of the trophic guilds was more variable in the kelp forest than the urchin barrens. Further, primary producer, grazer and predator assemblages were each most variable at the smallest spatial scale and least variable at the largest spatial scale within the kelp forests, but they followed the opposite pattern in the urchin barrens. In contrast, filter feeder assemblages were most variable at the largest spatial scale in kelp forests but at the smallest spatial scales in urchin barrens. However, given that many of the benthic filter feeders in this study (i.e., sponges and tunicates) exhibit indeterminate growth, a wide range in body sizes, and encrusting morphologies, we believe that estimates of variability based on density are insufficient to properly describe their abundances or spatial variation. Consequently, when abundances were estimated based biomass data, variability in filter feeder assemblages followed the same patterns observed for the whole communities and the for other feeding guilds; they were each most variable at the smallest scale and least variable at the largest scale in the kelp forests, but followed the opposite pattern in the urchin barrens. We believe that the differences in filter feeder scaling patterns between the two data types are likely not a sampling artifact but rather because biomass is the better measure of filter-feeder abundance and variation. Indeed, other studies have found discrepancies in community structure and distribution patterns for sponges when examining both density and biomass^71,72^. Likewise, the grazers were dominated by gastropods, whose heavy calcium carbonate shells may have dominated their biomass estimates, and thus were likely better described by their densities. In other words, the two sampling methods assess different taxa in the communities and this is important to how these communities are organized spatially. Consequently, we propose that each trophic guild followed the same scaling patterns as did the whole communities, and thus were likely affected by the same forcing factors. Specifically, they were each most variable at the smallest spatial scale within the kelp forests, which again is likely due to enhanced biological interactions^51,53,64^. As the kelp forests transitioned to urchin barrens and species diversity was reduced, the effects of these interactions became less important at larger spatial scales and the importance of physical factors and oceanographic control increased^35,62,73–75^.

Our findings are in agreement with previous studies in coastal marine systems that have found variability in the structure of biological communities to exist at multiple spatial scales^21,26,33–35^. These patterns are often due to different forcing factors that operate across a range of scales^21,32,33,54^. For example, variation in biological interactions can drive variation at small scale (< 10 m) patterns, while habitat heterogeneity can drive local scale (< 10 km) differences in community structure^33,76,77^. At mesoscales (10–100 km) or regional scales (> 100 s km), coastal marine communities may differ across oceanographic boundaries where multiple physical parameters change simultaneously^35,78,79^. This can have important consequences to our understanding of the relative importance of the factors that structure these communities, as small-scale biological interactions can mask the influence of large-scale oceanographic and climate factors^51–53^, while large-scale factors can mask the influence of small-scale biological interactions^33,62–64^. Here, we show that variation in Aleutian kelp forest communities generally follows a scaling pattern, which is similar to patterns observed for giant kelp, *Macrocystis pyrifera*, populations along the coasts of California, USA and Baja California, MEX^33,35^. There, as with our current study, the greatest amount of variability was observed at spatial scales that encompassed 10 s of meters (i.e., among samples within each kelp forest) compared to scales that encompassed 100 s to 1000 s of kilometers (i.e., among geographic regions). However, that scaling pattern was altered by large-scale changes in ocean temperature and wave intensity during the 1997–1998 ENSO event, which affected the kelp populations at large (100 s–1000 s km) spatial scales and masked patterns of small-scale variability^27^. Specifically, this resulted in a reduction in small-scale variability and a shift towards large-scale variability^33^. Given the Aleutian nearshore ecosystem is a top-down driven system where, in general, environmental variables play a small role in determining community structure and the presence of alternate stable states^56^, we suggest the effects of trophic downgrading in the Aleutian kelp forests were similar to the ENSO effects in the California and Mexico kelp populations in that they reduced overall spatial variability in community structure and shifted this variability from small scales (i.e., within forests) to large scales (among geographic regions), and propose that this occurred due to a shift in the relative importance of biological interactions towards oceanographic forcing.

Ecological theory predicts that global warming will increase the importance of foundation species in maintaining ecosystem function because they can ameliorate environmental stress^79–81^. In the Aleutian Archipelago, we have demonstrated that the trophic downgrading that has greatly eliminated foundational kelp species has also reduced variability in overall community structure and in the structure of trophic guilds. While functional redundancies among predators and herbivores can make diverse systems more stable^7,9^, simple food webs, such as those found in the Aleutian Archipelago, do not have the functional redundancies at higher trophic levels needed to maintain stability. Trophic rewilding (i.e., the restoration of apex predators) has been suggested for systems that have been downgraded^82^ and may be one way that Aleutian kelp forests and their spatial variability can be restored. However, this may also be problematic for the Aleutian ecosystem given that killer whales still may be hunting sea otters there. Further, the establishment of a successful sea urchin fishery (i.e. human predation) to reduce sea urchin numbers may also not be feasible because the urchins in these barrens are generally characterized as having small gonads of poor quality due to a lack of macroalgal food^83^. But, as ocean temperatures in this region continue to rise with climate change^84^, the possibility of urchin disease may increase^85,86^, which could overtake predation as the primary source of urchin mortality^87^ and result in substantial declines in urchin populations^88–90^. This can ultimately lead to a restoration of the ecosystem as seen in other areas of the world^80,88,91,92^. Regardless, we believe some mechanism of widespread urchin mortality may be necessary to return normal variability in ecosystem structure.

## Materials and methods

### Study region and sites

The Aleutian Archipelago is a volcanic mountain chain that extends approximately 1900 km over 25° of longitude and 4° of latitude. The islands separate the Bering Sea to the north from the Pacific Ocean to the south, with the eastern extent occurring at the Alaska Peninsula, USA and the western extent occurring at the Kamchatka Peninsula, RUS. Island sizes are variable (27–2500 km^2^), as is the extent of the continental shelf (i.e., the 200 m isobath) around these islands (10–100 km). Oceanographic passes separate many of the island groups and facilitate water exchange between the Bering Sea and Pacific Ocean^88^. Net water flow through the passes is northward, but the direction and water flow rate through these passes depend on pass width and depth^66,93^, which vary across the island chain^94^. Because of these conditions, the hydrodynamic environments around the different islands can vary.

We sampled nine islands spanning ~ 1230 km of the Aleutian Archipelago, from Attu to Chuginadak (Fig. 1), during two research cruises aboard the *R/V Oceanus* in July 2016 and June 2017. Specifically, we sampled Adak, Chugidinadak and Tanaga in 2016, and Amchitka, Atka, Attu, Kiska, Nizki, and Yunaska in 2017. Previous analyses of community data by our research group did not identify any differences in community structure between the two sample years^12,16^. Two 6–8 m deep sites belonging to each habitat type (kelp forest and urchin barren) were sampled at each island using SCUBA. Depending on location and accessibility, the sites within each island were separated by hundreds of meters to several kilometers. The kelp forests were identified as having canopy-forming kelp (*Eualaria fistulosa;* hereafter *Eualaria*) and a mixed sub-canopy comprised of kelps and other brown and red fleshy macroalgae. In contrast, the urchin barrens lacked nearly all canopy and sub-canopy fleshy macroalgae, but had an abundance of green sea urchins (*Strongylocentrotus* spp.) (see Metzger et al.^16^ for more detailed descriptions of community compositions).

### Community structure sampling methods

We estimated the density and biomass of benthic invertebrates and macroalgae occurring within each habitat type at each site. For this, all epibenthic organisms that occurred within ten haphazardly placed 0.25 m^2^ quadrats, except those strongly adhered to the substrate (e.g., barnacles and encrusting coralline algae), were scraped from the substrate and placed in fine mesh (< 1 mm) collection bags for shipboard processing. In addition, *Eualaria* sporophytes were counted within three haphazardly placed 10 m × 2 m swaths. Also along these swaths, all conspicuous and sparsely distributed large mobile invertebrates (e.g., sea stars, crabs, and large gastropods) whose densities were assumed to be less than 1.0 per 2.5 m^2^ (the total area covered by the quadrats in each site) were collected in fine mesh bags for shipboard processing. All collected organisms were identified to the lowest possible taxonomic level, counted if the taxon had discrete individuals, and weighed (to the nearest 0.005 kg) for biomass using hanging spring scales. Ambiguous or difficult to identify individuals were preserved in 10% formalin (for invertebrates) or pressed on herbarium paper (for algae) for later identification.

Following identification, the organisms were grouped according to their primary feeding method. Working with density and biomass based data separately, each taxon was assigned to one of four trophic guilds; namely primary producers, herbivores, filter feeders, or predators. Specifically, all macroalgae were classified as primary producers, and all ascidians, sponges, and cnidarians were classified as filter feeders. Taxa that also fed on detritus were classified according to their other primary feeding method (e.g., those that both grazed on algae and fed on detritus were classified as grazers, and those that were both predatory and fed on detritus were classified as predators). Taxa that were extremely rare (i.e., only a single individual occurred in one or two of our samples) were not included in this analysis.

### Community structure statistical analyses

All statistical analyses were done in Primer-E (Version 7) and R-Studio (Version 1.1.463). Quadrat and swath data were combined into density and biomass datasets within each habitat separately (kelp forests and urchin barrens). Consequently, our sampling method resulted in four discrete data sets; density and biomass estimates for organisms occurring in both kelp forests and sea urchin barrens. Density and biomass data were square root transformed prior to analysis to remove the effects of numerically dominant taxa. Four separate zero-inflated Bray–Curtis resemblance matrices assessing dissimilarities in community assemblages among quadrats within each site, between sites within each island, and among islands were generated; one for density-based data and one for biomass-based data within each of the two habitats. Variation in community structure was then evaluated among the different spatial scales within each of the two habitat types using four separate three-factor fully-nested PERMANOVAs (for each data type and habitat). Along with the PERMANOVAs, we used variance partitioning^59^ to compare patterns of variability among the different spatial scales and between the habitat types within each PERMANOVA model. Additionally, multivariate dispersions (i.e., variances in community assemblages) were determined for each spatial scale and within each habitat type using the MvDisp routine in Primer-E. Lastly, nMDS (nonmetric metric multidimensional scaling) plots were generated to visually display similarities in community assemblages at each spatial scale for each data type. The MDS axis values were imported into R-Studio where they were re-graphed with the exterior polygon points connected, which allowed us to evaluate the area encompassed by each habitat (i.e. convex hulls). Following analyses for the community assemblages, variation in the assemblage structures making up each feeding guild were similarly examined at each spatial scale using the MvDisp procedure in Primer-E as described above.

## Supplementary information

Supplementary Information
