# Supplementary material for: Trophic downgrading reduces spatial variability on rocky reefs
Source: Sci Rep. 2020 Oct 22;10:18079. doi: 10.1038/s41598-020-75117-2 (PMC7581756; doi:10.1038/s41598-020-75117-2)
Supplement: Supplementary file 1 — Supplementary Information [file 41598_2020_75117_MOESM1_ESM.pdf]

# Trophic downgrading reduces spatial variability on rocky reefs

Matthew S. Edwards[1,*], Brenda Konar[2]

**Supplemental material**

**Table S1.** Results of nested PERMANOVAs evaluating variation in community assemblages among the different spatial scales (among quadrats within each site, among sites within each island, and among islands) for A) kelp forests and B) urchin barrens based on abundance data, and for C) kelp forests and D) urchin barrens based on biomass data. For each analysis, the degrees of freedom weighted variance and percent of the total variation explained by each factor (i.e., magnitude of effects $\omega^2$) is given according to Graham and Edwards[57], as is the total underlying variation in each model. Prior to analysis, all data were square root transformed to down weigh the influence of overly abundant taxa.

**A. Kelp - Density**

| Source | df | SS | MS | Pseudo-F | P(perm) | Variance | $\omega^2$ |
|---|---|---|---|---|---|---|---|
| Island | 8 | 1.47E+05 | 18413 | 2.50 | 0.001 | 558.87 | 0.20 |
| Site(Island) | 9 | 66307 | 7367.4 | 4.56 | 0.001 | 582.11 | 0.21 |
| Quadrat(Site(Island)) | 160 | 2.58E+05 | 1614.4 | | | 1614.40 | 0.59 |
| Total | 177 | 4.06E+05 | | | | 2755.38 | |

**B. Barrens - Density**

| Source | df | SS | MS | Pseudo-F | P(perm) | Variance | $\omega^2$ |
|---|---|---|---|---|---|---|---|
| Island | 8 | 91619 | 11452 | 3.06 | 0.001 | 385.77 | 0.23 |
| Site(Island) | 9 | 33633 | 3737 | 3.70 | 0.001 | 272.83 | 0.16 |
| Quadrat(Site(Island)) | 162 | 1.63E+05 | 1008.7 | | | 1008.70 | 0.61 |
| Total | 179 | 2.89E+05 | | | | 1667.30 | |

**C. Kelp - Biomass**

| Source | df | SS | MS | Pseudo-F | P(perm) | Variance | $\omega^2$ |
|---|---|---|---|---|---|---|---|
| Island | 8 | 5193.7 | 649.21 | 2.58 | 0.068 | 20.10 | 0.20 |
| Site(Island) | 9 | 2267 | 251.89 | 4.10 | 0.001 | 19.27 | 0.19 |
| Quadrat(Site(Island)) | 160 | 9832.9 | 61.46 | | | 61.46 | 0.61 |
| Total | 177 | 17293.6 | | | | 100.83 | |

**D. Barren - Biomass**

| Source | df | SS | MS | Pseudo-F | P(perm) | Variance | $\omega^2$ |
|---|---|---|---|---|---|---|---|
| Island | 8 | 15846 | 1980.70 | 8.52 | 0.003 | 87.41 | 0.65 |
| Site(Island) | 9 | 2092.4 | 232.49 | 8.66 | 0.001 | 20.56 | 0.15 |
| Quadrat(Site(Island)) | 162 | 4349.1 | 26.85 | | | 26.85 | 0.20 |
| Total | 179 | 22287.5 | | | | 134.82 | |
